# Is Brief Exposure to Green Space in School the Best Option to Improve Attention in Children?

**DOI:** 10.3390/ijerph18147484

**Published:** 2021-07-13

**Authors:** Asier Anabitarte, Gonzalo García-Baquero, Ainara Andiarena, Nerea Lertxundi, Nerea Urbieta, Izaro Babarro, Jesús Ibarluzea, Aitana Lertxundi

**Affiliations:** 1 Preventive Medicine and Public Health, University of the Basque Country (UPV/EHU), Barrio Sarriena s/n, 48490 Leioa, Spain; aitana.lertxundi@ehu.eus; 2 Biodonostia, Environmental Epidemiology and Child Development Group, Paseo Doctor Begiristain s/n, 20014 Donostia-San Sebastian, Spain; ggbmoneo@ciberesp.es (G.G.-B.); ainara.andiarena@ehu.eus (A.A.); nerea.lertxundi@ehu.eus (N.L.); n-urbietamacazaga@euskadi.eus (N.U.); izaro.babarro@ehu.eus (I.B.); mambien3-san@euskadi.eus (J.I.); 3 Consortium for Biomedical Research in Epidemiology and Public Health (CIBERESP), Instituto de Salud Carlos III, Av. Monforte de Lemos, 3–5. Pabellón 11. Planta 0, 28029 Madrid, Spain; 4 Faculty of Psychology, University of the Basque Country (UPV/EHU), Av. Tolosa 70, 20018 Donostia-San Sebastian, Spain; 5 Ministry of Health of the Basque Government, SubDirectorate for Public Health and Addictions of Gipuzkoa, Av. Navarra 4, 20013 Donostia-San Sebastián, Spain

**Keywords:** attention network test, natural spaces, mental health, ReML

## Abstract

The positive effects of Green Spaces on health are thought to be achieved through the mechanisms of mitigation, instoration and restoration. One of the benefits of Green Spaces may be the restoration of attention and so the objective of this research is testing empirically whether exposure to a green environment improves attention in school children. For so doing, we first used a split-unit statistical design in each of four schools, then combined the primary results via meta-analysis. The Attention Network Test (ANT) was used to measure attention before and after exposure and a total of 167 seven-year-old students participated in the experiments. Overall, our experimental results do not support the hypothesis that students’ exposure to activities in green vs. grey spaces affected their performance in ANT. This was so despite the fact that neither age nor gender biases have been detected and despite that our experiments have been proved to be sufficiently statistically powerful. It would be advisable to consider air pollution and noise. We also recommend that participants attend the experiment with mental exhaustion to maximize the ability to detect significant changes.

## 1. Introduction

In an increasingly urban world, with almost 75% of the European population living in urban areas [1], urban planners and public health professionals need to work together [2] since various environmental exposures and lifestyles detrimental to health can be found in cities [3]. Natural spaces such as parks, gardens, urban trees, etc. are becoming increasingly important for improving the quality of life and health of citizens [4,5] and according to the World Health Organization (WHO), Urban Green Spaces are beneficial for health [6]. Therefore, and with the sustainable development goals set by the UN (SDG3 and SDG11), it is important that the contributions of Green Spaces to cities are taken into account [7].

According to scientific evidence, the mechanisms that explain the positive effects of Green Spaces on health are through mitigation, instoration and restoration. Different authors agree that Green Spaces can have positive effects on health through mitigation, instoration and restoration [8,9]. They mitigate air pollution and noise levels [10,11,12] and are able to regulate temperature by preventing possible heat waves [13,14]. In addition, their instoration role comes from the contribution of new resources because Green Spaces promote physical activity [15,16,17] and the relationship between neighbours by strengthening social cohesion [18,19]. In regards to the restorative role, there are two theories: on the one hand, the stress reduction theory (SRT) and, on the other hand, the attention restorative theory (ART). The SRT states that Green Spaces are a source of positive emotions while blocking negative emotions and therefore reducing stress levels [20,21]. According to ART, Green Spaces offer a space to attract and recover attention effortlessly and suppress neurocognitive load [22,23]. ART suggests that natural spaces would stimulate involuntary attentional processes and therefore voluntary attentional processes would be restored. Living in environments that reduce the restorative capacity of attention, mainly in childhood, would negatively affect learning, the academic process and the development of social functions [24,25]. Those are the reasons why it is important to provide young students with care restoration areas [26].

There are few observational studies on the potential effect of Green Spaces in urban environments and their effect on attention. Depending on the type of exposure, two types of studies have been carried out: observational studies with long time exposure and interventional studies with brief exposure. Among the first ones, we will mention the study of Dadvand et al. [27] carried out with 2593 children between 7 and 10 years old living in the city of Barcelona. They focused on the beneficial role of green environments in cognitive functions, including attention. Specifically, they wanted to analyse the effects Green Spaces’ exposure had on superior working memory and attention. They found that the greater the amount of green space exposure both at home and at school, and the lower the levels of air pollution, the better the children’s cognitive performance. Additionally, also in Barcelona Amoly et al. [28], analysed the greenness of the city’s school yards and the environment of 2111 school children’s (7–10 years) homes and found a statistically significant inverse relationship between playtime in Green Spaces and inattention scores. Several authors point out as a limitation the lack of existence of a large number of longitudinal studies and therefore, that one cannot speak of an association [27,28,29].

Interventional studies related to Green exposure perform the analysis using brief exposure instead of long time exposure. One of the first studies was by Kuo and Faber [30], in which they exposed 452 children of 5–18 years of age with ADHD (Attention Deficit and Hyperactivity Disorder) symptomatology to an activity in nature, observing an improvement in ADHD symptoms in those who performed the activities exposed to Green Spaces several times compared to those who performed the activities in an indoor or outdoor built space.

Schutte et al. [31] found improvements in attention and working memory tasks for those who took a nature walk in a sample of 33 children aged 4–5 and 34 children aged 7–8. According to the results of the study by Stevenson et al. [32], performed with 33 Danish students aged 12, walking 30 min in a natural environment—compared to walking a similar time in an urban environment—is beneficial for children’s attention, being associated with faster and more stable responses on the ANT (Attention Network Test) test. However, Hartig et al. [33] observed no difference between the group exposed to natural environments and the control group, exposed to urban spaces, for restoration of attention.

The present study aims to assess whether 60-min of exposure, involving a short walk, an activity and having a mid-morning snack, to a green environment improves attention compared to another environment (grey spaces) in the general population of seven-year-olds.

## 2. Materials and Methods

### 2.1. Type of Study

This work has a quasi-experimental analytical epidemiological design. The exposure was defined as the environment where a programmed activity takes place: green space and grey space (a space without green environment). The assignment of the environment to each classroom (green/grey) was made randomly in each school. Green Spaces consist of an open green space with trees bigger than 5000 m^2^, while grey spaces are paved squares or schoolyards.

This study was carried out after obtaining the approval of the Ethical Committee of the Department of Health of the Basque Government as well as with the approval of the schools and with the signed informed consent of the parents or guardians of the children.

### 2.2. Study Area

The study area focuses on schools located in two coastal municipalities of Gipuzkoa, Zarautz and Donostia-San Sebastian, Basque Country, northern Spain. These municipalities are located 20 km from each other. Donostia-San Sebastian is the capital of Gipuzkoa, while Zarautz is the fifth largest municipality in the province by population, 23,223 inhabitants in Zarautz and 186,665 inhabitants in the capital (INE, 2018). They are two municipalities with a very defined urban network; Donostia-San Sebastian being the capital, has very important road axes in addition to urban traffic. At the same time, two busy roads cross the municipalities of Zarautz and Donostia, the AP-8 highway, which connects Donostia-San Sebastian with the other two provincial capitals of the Basque Country with an average daily traffic intensity of 44,890 vehicles in Zarautz and 49,244 vehicles in Donostia, and, on the other hand, the N-634 road, which crosses the entire coast of Gipuzkoa with an average daily traffic intensity of 7235 vehicles at the closest seating station to Zarautz [34].

Of the four schools, two of the schools (School 1 and 2) have a green space less than 300 m away, as recommended by the WHO [6], while the other two (School 3 and 4) have a green space more than 300 m away.

### 2.3. Sample

The participants were 167 children of 7 years old ($\bar{x}$ = 6.8, SD = 0.3) distributed in 4 schools, 3 in Zarautz and 1 in Donostia-San Sebastián. The four schools have similar characteristics in terms of the socioeconomic level of the students, all above the MEDEA 4–5 deprivation index, which allows them to be classified as areas of a high socioeconomic level (http://www.proyectomedea.org, accessed on 18 August 2020). None of the study participants had a previous diagnosed ADHD symptomatology. Among the participants, 7 children had to be excluded from the study because they did not meet the criteria for inclusion in the test of care used (a score of less than 90 in the test scores variable) (proyectoinma.org accessed on 10 July 2020).

### 2.4. Attention Network Test (ANT)

Attention was evaluated using the children’s version of the neuropsychological Attention Network Test (ANT) [35]. The children’s version of ANT [36] was designed to measure attention in children aged 6 to 10. To make it more user-friendly, the arrows in the adult version were replaced with fish with an arrow inside. Children should look at the fish in the middle of a row of 5 fish and use the arrows on the keyboard to indicate the same direction as that fish. For each correct hit the children will have a positive hearing reinforcement (¡Woo-hoo!) [25]. This test has been used in several studies measuring the effect of Green Spaces on the attention of children of similar ages to the sample above [32,37,38].

The test was conducted in the children’s usual classroom. Computers were distributed in such a way that participants did not disturb each other and they were provided with headphones to listen to the auditory stimulus when responding correctly and to avoid possible sound discomfort.

Each classroom had 3–4 instructors and 19 children on average. All participants received the same instructions, which were agreed upon by all instructors. The first time-block was always for training and once the instructors checked that each child had understood how to do the test, the training ended and the test started, which consisted of 4 blocks of 32 tests each (128 tasks in total). 

Five different variables were obtained through the test. The variable “test scores” represents the score of the hits obtained; “accuracy” defines the accuracy, calculated as accuracy = test scores/(errors + test scores). The variable “Reaction time” marks the median time that the participants took to answer each test. One of the most interesting variables of the test, along with that of the scores, is the variable “Reaction time variability”, which indicates the variation in response time between the different tests of each participant. This variable has been related to inattention [37]. Finally, the “impulsivity” was calculated as (impulsivity = (Reaction time in correct responses − Reaction time in incorrect responses)), which is interpreted as the higher value being the less impulsive. This test does not allow us to know whether voluntary attention processes have been deactivated during the activity, but we understand that significant reductions in reaction time variability could be compatible with this change in the attentional system (voluntary-involuntary).

### 2.5. Procedure

Four schools were contacted and all of them gave their approval to carry out the study within their premises. The project started after obtaining the signed consent of the principals. First, the school was visited in the early morning to prepare the necessary material and to set up the computers. Then, participants entered classrooms at the time they normally start lessons, at 9 a.m., and each subject was placed in their respective computer with the ID assigned to them. The informed consent forms, signed by the parents or legal guardians and distributed earlier by the school, were collected and the explanation began. The participants were not informed in advance of the test they were to take. Only data belonging to those who gave their informed consent were analysed.

**Figure 1:**
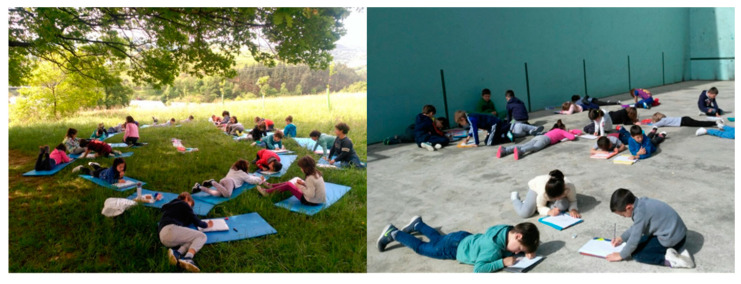
Participants in the green and grey spaces.

Once all the students were in place, it was explained to them how they should proceed for the correct realization of the practice. The ANT (Attention Network Test) was carried out for 20 min. Once the whole class finished the exercise (pre-intervention test), they went out to the school playground or street. Then, the class was divided into two groups: one went to a green space and the other to a grey space to perform the activity (Figure 1).

All the groups performed the same playful activity which consisted of playing a game and then having a mid-morning snack; altogether the activity lasted 60 min. Afterwards, they returned to the class and each participant went back to the same computer where they first performed the test, in order to repeat it. After the second test, the group’s experiment was concluded.

### 2.6. Power Analysis

A power analysis was carried out in order to calculate the sample size required for each outcome of the study. For this purpose, the R package BDEsize: Efficient Determination of Sample Size in Balanced Design of Experiments [39] was used. The defined parameters were: alpha = 0.05, effect size (exposure × time interaction) = 15%, obtaining statistical power of 85%. The sample size was different for each outcome: total score (*n* = 4 for each group), accuracy (*n* = 3 for each group), reaction time (*n* = 16 for each group) and reaction time variability (*n* = 22 for each group) and impulsivity (*n* = 664 for each group). In the case of impulsivity, due to its variability, a large sample size would be required. Therefore, the results obtained in the experiment should be interpreted with caution.

### 2.7. Description of the Experiments

A basic experiment was carried out in each of four schools, namely School 1, School 2, School 3 and School 4 (Supplementary Table S1), to test for the effect of the exposure to activities in green vs. grey spaces on the school students’ performance in multiple-choice computer-based tests (128 questions). Students (random factor <Subject>) were randomly assigned to each of two levels of the fixed factor <Exposure>, that is <grey> and <green> spaces. Five univariate responses were recorded in two occasions on each subject, one before applying the exposure (level <before> of the fixed factor <Time>) and another after applying the exposure (level <after> of the fixed factor <Time>). Student’s sex and age were also recorded.

### 2.8. Repeated Measures: Statistical Design and Model

Each basic experiment is to be understood as a split-unit (or split-plot) statistical design [40], where subjects represent the whole units, exposures represent the whole-unit treatments and time represents the split-unit treatment. The two measurements of each response taken on each subject are correlated although, there being just two repeated measures, we may assume equicorrelation [40] and, therefore, Cochran’s theorem [41] is satisfied, with the final consequence that F-tests are valid. This specific type of split-unit design is known as a repeated-measurement design and the corresponding statistical model may be written as follows [40,41,42]:

*Y*_*ijk*_ = *µ* + *τ*_*i*_ + *ε*_*ij*_ + *γ*_*k*_ + *(τγ)*_*ik*_ + *δ*_*ijk*_, (1)

Equation (1). where *Y_ijk_* is a given response to Exposure *i* (= 1, …, *t*) of subject (whole unit) *j* (= 1, …, *r*) at Time *k* (= 1, …, *g*); *µ* is the overall mean effect; *τ_i_* is the effect of Exposure *i* (i.e., the whole unit treatment: Subjects in Exposure); *ε_ij_* is the effect of Subject j in Exposure *i* (i.e., the whole unit error), where *ε_ij_* is independently and identically distributed as N(0, σ2ε); *γ_k_* is the effect of Time k (i.e., the split unit treatment); *(τγ)_ik_* is the interaction between Exposure *i* and Time *k*; and *δ_ijk_* is the experimental error (i.e., the split unit error: Time x Subjects in Exposure), where *δ_ijk_*, which is independent of *ε_ij_*, is assumed to be independently and identically distributed as N(0, σ2δ). Since Exposure has two levels (green and grey spaces) and since Time has two levels (before and after), *t* = 2 and *g* = 2; since different numbers of subjects were used in each school, *r* varies in each basic experiment as specified in Supplementary Table S1.

Thus, the effect of the exposure under a repeated-measurements design is measured by the term *(τγ)_ik_* in Equation (1), i.e., by the interaction between Exposure *i* and Time *k*. Since the goal of each of our basic experiments was to test for the effect of the exposure to activities in green vs. grey spaces on the school students’ performance in multiple-choice tests, we focused our analysis on said term.

### 2.9. Linear Mixed Effects Modelling: The Analysis of the Basic Experiments

Univariate responses from experimental settings may be analysed using the ANOVA approach [40], but we preferred the Restricted Maximum Likelihood (ReML) approach within the framework of linear mixed effects modelling [42,43]. This was so because the flexible ReML approach yields unbiased estimates for random terms in mixed models (such as the terms *ε_ij_* and *δ_ijk_* in Equation (1). Hence, for the purpose of analysing (i.e., for hypothesis testing and parameter estimation) all the responses in our basic experiments, according to Equation (1), we used function lme() of R package nlme [44] in R software v. 4.0.0 (R Foundation for Statistical Computing, Vienna, Austria) [45]. Model assumptions, as made explicit in Equation (1), were checked for each fitted model and variance function structures [46] were introduced, when needed, through the argument weights of lme().

### 2.10. Meta-Analysis: Combining the Results of the Basic Experiments

Once we quantified the effect of the exposure levels in each basic experiment (as measured by the term *(τγ)_ik_* in Equation (1), we combined the available evidence from School 1, School 2, School 3 and School 4 experiments using the meta-analysis methodology [47]. For this purpose, we used the function metagen() of the R package meta [48], applying the generic inverse variance method [49] for pooling the available data of each of the five response variables.

For more information about the methods, see Supplementary Material.

## 3. Results

As can be seen in Table 1, the maximum score obtained in score was 128, and on average the children failed eight (120 on average in score), with a minimum score of 91. The average value of the accuracy of the test was high, close to 1. As for the reaction time, in ms, the average was 924 ms and the standard deviation was 194 ms. A similar coefficient of variation is observed in both variables, c. ~23% variation around the mean.

Because neither age nor gender biases were detected (Supplementary Table S1), we concluded that the randomization of students to exposure levels was correct. The only strong correlation between responses occurred between test score and accuracy (0.603) (Supplementary Table S2), which is logical.

The Exposure × Time interaction effect (Table 2), the focus of our inference, was not found to be significant for any combination of response and school. Thus, we did not find evidence that students’ exposure to activities in green vs. grey spaces affected their performance in multiple-choice tests. The same is true for the main effect corresponding to Exposure (Table 2), which, again, suggests that, in our repeated-measures designs, the randomization of students to exposure levels was correct. By contrast, the main Time effect was significant in most schools for reaction time and variability in reaction time (Table 2). These main Time effects are negative, indicating that students’ reaction time was lower after being exposed to activities in green and grey spaces than before, regardless of the exposure. More information about the results can be found in Supplementary Material, Figures S1–S5.

**Figure 2:**
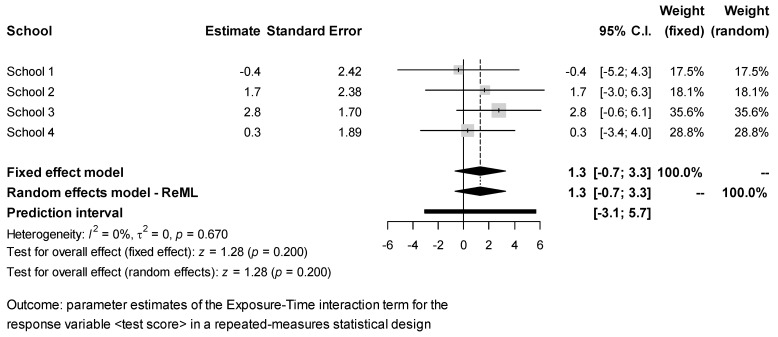
Meta-analysis for test response test score.

**Figure 3:**
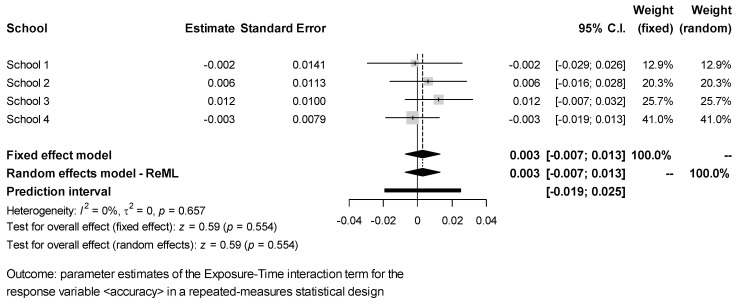
Meta-analysis for test response accuracy.

**Figure 4:**
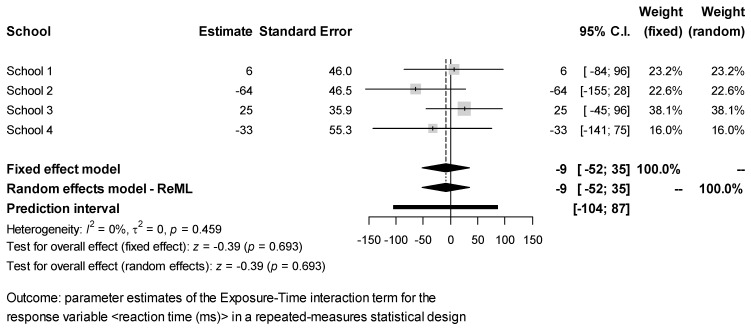
Meta-analysis for test response reaction time.

**Figure 5:**
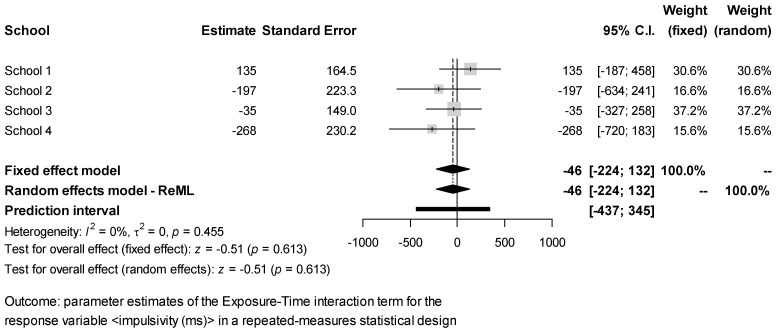
Meta-analysis for test response impulsivity.

**Figure 6:**
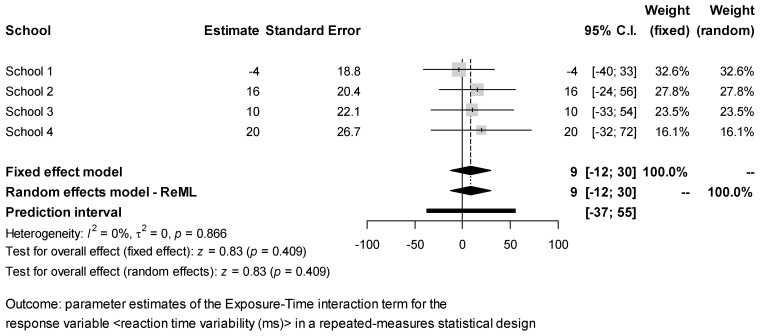
Meta-analysis for test response reaction time variability.

The estimates of the Exposure × Time interaction effect for each response obtained in the different schools were combined via meta-analysis. The results (Figure 2, Figure 3, Figure 4, Figure 5 and Figure 6) were found to be unbiased despite the small number of studies (Supplementary Table S3). However, they do not support the idea that students’ exposure to activities in green vs. grey spaces affected their performance in attentional multiple-choice tests, for each confidence and prediction interval includes the value zero. Besides, because in every case *τ*_2_ = 0, the fixed effects model was found to be sufficient, thereby suggesting that the observed variability in the schools’ estimates can be explained simply by the expected estimator variance. Information about the funnel plots can be found in Supplementary Material, Figure S6.

## 4. Discussion

The aim of this work was to study the relationship between carrying out an activity in a short period in a green space and the restoration of attention in seven-year-old children. Although no interaction was found between exposure and time, after the activity—regardless of exposure—student responses improved in reaction time and reaction time variability. Previous studies have shown that long time exposure to green space can influence restoration of attention [37]; however, this study examined the effect that green space can have on restoring attention but found no effect. This result may be due to the fact that the study area is located in very green areas itself, as a consequence of being in the Atlantic climate: a temperate-humid climate, showing moderate temperatures and being very rainy without a dry season. This combination favours the proliferation of vegetation [50]. In contrast, Dadvand’s [37] study area is a Mediterranean climate zone, with hot and dry summers and mild and rainy winters. In conclusion, there are fewer green areas compared to the Atlantic climate zones. In addition, other studies have found that more urbanised areas or city centres benefit more from the health effects of Green Spaces compared to rural areas, which may be due to the increased pace of life and stress that can occur in large cities [51,52]. Our study area consists of Zarautz, a town of around 23,000 inhabitants and Donostia, a city of around 186,000 inhabitants, which is small and surrounded by mountains.

Regarding the meta-analysis, no significant results were found for the test answers. No differences were found between the groups that performed the activity in the green space near the school and those that performed it in the grey space near their school. It should be noted that inter-individual variability could explain a great part of the variance, in many cases exceeding 50%. Although in the present study information was collected about gender and the same protocol was followed when carrying out the intervention to avoid variability in the estimates, it is estimated that, even so, the inter-individual weight is very high.

In addition, schools are usually located next to roads with heavy traffic [53]; this reality also exists with the four schools in the study. This makes it difficult to select noise-free spaces to develop the experiment in an environment free of negative exposures; therefore, results may be influenced by some environmental stressors such as noise, since all schools are located in noise zones that are higher than those dictated by legislation [54], which could interfere with the participants’ ability to restore the attention, since according to the literature noise has a negative influence.

Some studies have linked the presence of Green Spaces with decreased noise levels, although there is not yet consistent evidence [55]; noise has been observed to have a causal relationship in inattention and affect cognitive development [56]. A study around Munich airport found a cause–effect relationship between environmental noise and cognitive skills among children. In the same study, groups exposed to an airport (old and new Munich airport) were compared with control groups, groups not exposed to aerial noise and with the same sociodemographic characteristics as the groups of those exposed. The groups exposed to airport noise have a longer reaction time in the attention test. When the old airport was closed, the group that had experienced such exposure improved in reading and long-term memory scores. In addition, the exposed group had worse reading scores two years after the new airport became operational, compared with scores one year after the new airport became operational, suggesting that there may be a cumulative effect of noise exposure [57].

In another study, Zhang, Kang, & Kang [58] analysed the restorative capacity of an urban natural environment but compared different groups exposed to three sound scenarios. They found that the group exposed to an urban natural environment with a natural sound environment had a greater restorative capacity than those exposed to traffic noise.

The idea that noise, as well as air pollution, interferes with restoring attention and that Green Spaces mitigate these exposures follows the model of Markevych et al. [9] and Hartig et al. [8]. With what was mentioned before, it can be concluded that not measuring exposure to noise as well as not considering more individual variables has been a limitation of this study.

Finally, an hour of activity in the Green Spaces may not have been enough time to appreciate improvements in attention and, therefore, the minimum time to consider in relation to attention is something to take into account in future studies. Several studies claim to see differences between exposed and unexposed groups with activities or walks of 20–30 min [32,59,60], however, a review of minimum time in nature for a positive impact found only three studies related to attention; all three studies designed time periods in Green Spaces of 50 min. One of them observed an improvement in attention, another observed no association and the last one observed worse scores in the attention test [61].

## 5. Conclusions

According to the results obtained, the main hypothesis that carrying out a short-term activity in a green space is restorative of attention is not fulfilled. However, it is recommended to continue analysing the relationship between Green Spaces and restoration of attention, taking into consideration for the analysis variables such as noise and air pollution as well as individual variables.

## Figures and Tables

**Table 1:** Descriptive statistics (mean of pre-post) for the five responses considered in this research work.

Schools 1, 2, 3 and 4									
Variable	n	Minimum	Maximum	Mean	StDev	Median	*Q*1	*Q*3	*IQR*
Test score	319	91.0	128.0	119.7	6.6	121.0	117.0	124.0	7.0
Test accuracy	319	0.823	1.000	0.967	0.032	0.976	0.957	0.992	0.035
Reaction time (ms)	319	527.0	1584.0	923.9	194.2	898.0	789.8	1044.5	254.8
Impulsivity (ms)	319	−1593.5	1406.0	117.2	447.9	71.5	−106.3	247.3	353.5
Variability in reaction time (ms)	319	112.4	485.1	329.5	73.6	336.8	287.2	375.6	88.4

**Table 2:** Hypothesis testing for the five response variables considered in the experiment.

Response is Test Score			School 1		School 2		School 3		School 4	
**Source**	**df (num)**	**df (den)**	***F*-value**	***p*-value**	***F*-value**	***p*-value**	***F*-value**	***p*-value**	***F*-value**	***p*-value**
(Intercept)	1	40	15611.40	<0.001	26003.36	<0.001	22164.02	<0.001	22581.18	<0.001
Exposure	1	40	0.05	0.834	0.64	0.431	0.56	0.456	0.40	0.534
Time	1	40	0.86	0.360	0.12	0.737	5.01	0.029	4.18	0.051
Exposure × Time	1	40	0.03	0.864	0.49	0.490	2.65	0.109	0.03	0.876
**Response is accuracy**			**School 1**		**School 2**		**School 3**		**School 4**	
**Source**	**df (num)**	**df (den)**	***F*-value**	***p*-value**	***F*-value**	***p*-value**	***F*-value**	***p*-value**	***F*-value**	***p*-value**
(Intercept)	1	40	42300.24	<0.001	95594.79	<0.001	83821.45	<0.001	49778.72	<0.001
Exposure	1	40	0.44	0.509	1.34	0.257	0.98	0.326	0.59	0.448
Time	1	40	0.04	0.833	1.35	0.255	2.11	0.151	11.07	0.003
Exposure × Time	1	40	0.01	0.910	0.31	0.584	1.50	0.226	0.14	0.712
**Response is reaction time**			**School 1**		**School 2**		**School 3**		**School 4**	
**Source**	**df (num)**	**df (den)**	***F*-value**	***p*-value**	***F*-value**	***p*-value**	***F*-value**	***p*-value**	***F*-value**	***p*-value**
(Intercept)	1	40	1132.50	<0.001	737.58	<0.001	1733.15	<0.001	1210.86	<0.001
Exposure	1	40	0.64	0.429	1.48	0.234	0.02	0.896	0.68	0.416
Time	1	40	51.67	<0.001	22.50	<0.001	16.38	0.000	17.84	<0.001
Exposure × Time	1	40	0.02	0.900	1.87	0.182	0.50	0.484	0.36	0.555
**Response is impulsivity**			**School 1**		**School 2**		**School 3**		**School 4**	
**Source**	**df (num)**	**df (den)**	***F*-value**	***p*-value**	***F*-value**	***p*-value**	***F*-value**	***p*-value**	***F*-value**	***p*-value**
(Intercept)	1	40	3.05	0.088	10.98	0.003	5.89	0.018	0.95	0.339
Exposure	1	40	1.47	0.233	1.88	0.181	0.01	0.908	2.69	0.113
Time	1	40	2.81	0.101	7.07	0.013	1.51	0.224	0.88	0.357
Exposure × Time	1	40	0.68	0.415	0.78	0.386	0.05	0.818	1.36	0.254
**Response is reaction time variability**			**School 1**		**School 2**		**School 3**		**School 4**	
**Source**	**df (num)**	**df (den)**	***F*-value**	***p*-value**	***F*-value**	***p*-value**	***F*-value**	***p*-value**	***F*-value**	***p*-value**
(Intercept)	1	40	1500.11	<0.001	633.75	<0.001	1457.69	<0.001	892.34	<0.001
Exposure	1	40	0.05	0.824	0.38	0.543	2.15	0.148	0.21	0.649
Time	1	40	3.27	0.078	1.88	0.181	8.15	0.006	4.29	0.048
Exposure × Time	1	40	0.04	0.849	0.60	0.444	0.22	0.643	0.56	0.462

## Data Availability

Not applicable.

## Supplementary Materials

The following are available online at https://www.mdpi.com/article/10.3390/ijerph18147484/s1, Table S1: Summary of the experimental layout, together with the number of subjects employed in each experiment (school). Table S2: Kendall’s rank correlation tau across schools, together with tests of the null hypothesis that (true) tau is equal to 0. Table S3: Egger’s test. Figure S1: Test score before and after experimental exposure to activities in grey and green spaces (fitted relationship). Figure S2: Test accuracy before and after experimental exposure to activities in grey and green spaces (fitted relationship). Figure S3: Reaction time (ms) before and after experimental exposure to activities in grey and green spaces (fitted relationship). Figure S4: Impulsivity (ms) before and after experimental exposure to activities in grey and green spaces (fitted relationship). Figure S5: Reaction time variability (ms) before and after experimental exposure to activities in grey and green spaces (fitted relationship). Figure S6: Funnel plots for the meta-analysis reported in Figure 1, Figure 2, Figure 3, Figure 4 and Figure 5.

## References

[1] EEA (2020). Urban Adaptation in Europe: How Cities and Towns Respond to Climate Change.

[2] Duhl L.J., Sanchez A.K. (1999). Healthy Cities and the City Planning Process: A Background Document on Links between Health and Urban Planning.

[3] Khreis H., Van Nunen E., Mueller N., Zandieh R., Nieuwenhuijsen M.J. (2017). Commentary: How to create healthy environments in cities. Epidemiology.

[4] Nieuwenhuijsen M., Khreis H. (2019). Integrating Human Health into Urban and Transport Planning.

[5] Fong K., Hart J.E., James P. (2018). A review of a epidemiologic studies on greenness and health: Updated Literature through 2017. Curr. Environ. Health Rep..

[6] Egorov A.I., Mudu P., Braubach M., Martuzzi M., WHO Regional Office for Europe (2016). Urban Green Spaces and Health.

[7] United Nations (2020). The Sustainable Development Goals Report.

[8] Hartig T., Mitchell R., de Vries S., Frumkin H. (2014). Nature and Health. Annu. Rev. Public Health.

[9] Markevych I., Schoierer J., Hartig T., Chudnovsky A., Hystad P., Dzhambov A.M., de Vries S., Triguero-Mas M., Brauer M., Nieuwenhuijsen M.J. (2017). Exploring pathways linking greenspace to health: Theoretical and methodological guidance. Environ. Res..

[10] David Suzuki Foundation (2015). The Impact of Green Space on Heat and Air Pollution in Urban Communities: A Meta-Narrative Systematic Review.

[11] Nowak D.J., Hirabayashi S., Bodine A., Greenfield E. (2014). Tree and forest effects on air quality and human health in the United States. Environ. Pollut..

[12] Van Renterghem T., Forssén J., Attenborough K., Jean P., Defrance J., Hornikx M., Kang J. (2015). Using natural means to reduce surface transport noise during propagation outdoors. Appl. Acoust..

[13] Weng Q., Yang S. (2004). Managing the adverse thermal effects of urban development in a densely populated Chinese city. J. Environ. Manag..

[14] Bowler D.E., Buyung-Ali L., Knight T.M., Pullin A.S. (2010). Urban greening to cool towns and cities: A systematic review of the empirical evidence. Landsc. Urban Plan..

[15] Almanza E., Jerrett M., Dunton G., Seto E., Ann Pentz M. (2012). A study of community design, greenness, and physical activity in children using satellite, GPS and accelerometer data. Health Place.

[16] McCormack G.R., Rock M., Toohey A.M., Hignell D. (2010). Characteristics of urban parks associated with park use and physical activity: A review of qualitative research. Health Place.

[17] Mytton O.T., Townsend N., Rutter H., Foster C. (2012). Green space and physical activity: An observational study using Health Survey for England data. Health Place.

[18] Kuo F.E., Sullivan W.C., Coley R.L., Brunson L. (1998). Fertile Ground for Community: Inner-City Neighborhood Common Spaces. Am. J. Community Psychol..

[19] Kemperman A., Timmermans H. (2014). Green spaces in the direct living environment and social contacts of the aging population. Landsc. Urban Plan..

[20] Ulrich R.S. (1983). Aesthetic and Affective Response to Natural Environment. Behavior and the Natural Environment.

[21] Ulrich R.S., Simons R.F., Losito B.D., Fiorito E., Miles M.A., Zelson M. (1991). Stress recovery during exposure to natural and urban environments. J. Environ. Psychol..

[22] Kaplan S. (1995). The restorative benefits of nature: Toward an integrative framework. J. Environ. Psychol..

[23] Kaplan R., Kaplan S. (1989). The Experience of Nature: A Psychological Perspective.

[24] Spira E.G., Fischel J.E. (2005). The impact of preschool inattention, hyperactivity, and impulsivity on social and academic development: A review. J. Child Psychol. Psychiatry.

[25] Suades-González E., Forns J., García-Esteban R., López-Vicente M., Esnaola M., Álvarez-Pedrerol M., Julvez J., Cáceres A., Basagaña X., López-Sala A. (2017). A Longitudinal Study on Attention Development in Primary School Children with and without Teacher-Reported Symptoms of ADHD. Front. Psychol..

[26] Vanaken G.-J., Danckaerts M. (2018). Impact of Green Space Exposure on Children’s and Adolescents’ Mental Health: A Systematic Review. Int. J. Environ. Res. Public Health.

[27] Dadvand P., Nieuwenhuijsen M.J., Esnaola M., Forns J., Basagaña X., Alvarez-Pedrerol M., Rivas I., López-Vicente M., De Pascual M.C., Su J. (2015). Green spaces and cognitive development in primary schoolchildren. Proc. Natl. Acad. Sci. USA.

[28] Amoly E., Dadvand P., Forns J., López-Vicente M., Basagaña X., Julvez J., Alvarez-Pedrerol M., Nieuwenhuijsen M.J., Sunyer J. (2015). Green and blue spaces and behavioral development in barcelona schoolchildren: The BREATHE project. Environ. Health Perspect..

[29] de Keijzer C., Gascon M., Nieuwenhuijsen M.J., Dadvand P. (2016). Long-Term Green Space Exposure and Cognition across the Life Course: A Systematic Review. Curr. Environ. Health Rep..

[30] Kuo F.E., Faber Taylor A. (2004). A potential natural treatment for attention-deficit/hyperactivity disorder: Evidence from a national study. Am. J. Public Health.

[31] Schutte A.R., Torquati J.C., Beattie H.L. (2017). Impact of Urban Nature on Executive Functioning in Early and Middle Childhood. Environ. Behav..

[32] Stevenson M.P., Dewhurst R., Schilhab T., Bentsen P. (2019). Cognitive restoration in children following exposure to nature: Evidence from the attention network task and mobile eye tracking. Front. Psychol..

[33] Hartig T., Mang M., Evans G.W. (1991). Restorative Effects of Natural Environment Experiences. Environ. Behav..

[34] Diputación Foral de Gipuzkoa (2019). Información de Aforos en Las Carreteras de Gipuzkoa.

[35] Fan J., McCandliss B.D., Sommer T., Raz A., Posner M.I. (2002). Testing the Efficiency and Independence of Attentional Networks. J. Cogn. Neurosci..

[36] Rueda M.R., Fan J., McCandliss B.D., Halparin J.D., Gruber D.B., Lercari L.P., Posner M.I. (2004). Development of attentional networks in childhood. Neuropsychologia.

[37] Dadvand P., Tischer C., Estarlich M., Llop S., Dalmau-Bueno A., López-Vicente M., Valentín A., de Keijzer C., Fernández-Somoano A., Lertxundi N. (2017). Lifelong Residential Exposure to Green Space and Attention: A Population-based Prospective Study. Environ. Health Perspect..

[38] Forns J., Esnaola M., López-Vicente M., Suades-González E., Alvarez-Pedrerol M., Julvez J., Grellier J., Sebastián-Gallés N., Sunyer J. (2014). The n-back test and the attentional network task as measures of child neuropsychological development in epidemiological studies. Neuropsychology.

[39] Bin Lim Y., Hee Chung J. (2019). BDEsize: Efficient Determination of Sample Size in Balanced Design of Experiments, R Package Version 1.2. https://cran.r-project.org/web/packages/BDEsize/BDEsize.pdf.

[40] Casella G. (2008). Statistical Design.

[41] Cochran W.G. (1934). The distribution of quadratic forms in a normal system, with applications to the analysis of covariance. Math. Proc. Camb. Philos. Soc..

[42] Pinheiro J., Bates D. (2000). Mixed-Effect Models in S and S-Plus.

[43] Bolker B.M., Brooks M.E., Clark C.J., Geange S.W., Poulsen J.R., Stevens M.H.H., White J.S.S. (2009). Generalized linear mixed models: A practical guide for ecology and evolution. Trends Ecol. Evol..

[44] Pinheiro J., Bates D., DebRoy S.S., Sarkar D. (2018). Nlme: Linear and Nonlinear Mixed Effects Models. https://rdrr.io/cran/nlme/.

[45] R Core Team (2020). R: A Language and Environment for Statistical Computing.

[46] Davidian M., Giltinan D.M. (1995). Nonlinear models for repeated measurement data: An overview and update. J. Agric. Biol. Environ. Stat..

[47] Higgins J.P., Green S. (2008). Cochrane Handbook for Systematic Reviews of Interventions.

[48] Balduzzi S., Rücker G., Schwarzer G. (2019). How to perform a meta-analysis with R: A practical tutorial. Evid. Based Ment. Health.

[49] Borenstein M., Hedges L.V., Higgins J.P.T., Rothstein H.R. (2010). A basic introduction to fixed-effect and random-effects models for meta-analysis. Res. Synth. Methods.

[50] Gobierno Vasco Clasificación de Territorios Climáticos. https://www.euskadi.eus/gobierno-vasco/contenidos/informacion/cla_clasificacion/es_7264/es_cliclasificacion.html.

[51] Mitchell R., Popham F. (2007). Greenspace, urbanity and health: Relationships in England. J. Epidemiol. Community Health.

[52] Engemann K., Pedersen C.B., Arge L., Tsirogiannis C., Mortensen B., Svenning J.-C. (2019). Residential green space in childhood is associated with lower risk of psychiatric disorders from adolescence into adulthood. Proc. Natl. Acad. Sci. USA.

[53] European Environment Agency (2018). Unequal Exposure and Unequal Impacts: Social Vulnerability to Air Pollution, Noise and Extreme Temperatures in Europe.

[54] Goverment B. (2012). Decreto 213/2012, de 16 de octubre, de contaminación acústica de la Comunidad Autónoma del País Vasco. Bol. País Vasco.

[55] Gidlöf-Gunnarsson A., Öhrström E. (2007). Noise and well-being in urban residential environments: The potential role of perceived availability to nearby green areas. Landsc. Urban Plan..

[56] Klatte M., Bergström K., Lachmann T. (2013). Does noise affect learning? A short review on noise effects on cognitive performance in children. Front. Psychol..

[57] Hygge S., Evans G.W., Bullinger M. (2002). A prospective study of some effects of aircraft noise on cognitive performance in schoolchildren. Psychol. Sci..

[58] Zhang Y., Kang J., Kang J. (2017). Effects of Soundscape on the Environmental Restoration in Urban Natural Environments. Noise Health.

[59] Faber Taylor A., Kuo F.E. (2009). Children with attention deficits concentrate better after walk in the park. J. Atten. Disord..

[60] Gidlow C.J., Jones M.V., Hurst G., Masterson D., Clark-Carter D., Tarvainen M.P., Smith G., Nieuwenhuijsen M. (2016). Where to put your best foot forward: Psycho-physiological responses to walking in natural and urban environments. J. Environ. Psychol..

[61] Meredith G.R., Rakow D.A., Eldermire E.R.B., Madsen C.G., Shelley S.P., Sachs N.A. (2020). Minimum Time Dose in Nature to Positively Impact the Mental Health of College-Aged Students, and How to Measure It: A Scoping Review. Front. Psychol..

