# Supplementary material for: Is Brief Exposure to Green Space in School the Best Option to Improve Attention in Children?
Source: Int J Environ Res Public Health. 2021 Jul 13;18(14):7484. doi: 10.3390/ijerph18147484 (PMC8304383; doi:10.3390/ijerph18147484)
Supplement: Supplementary file 1 [file ijerph-18-07484-s001.zip › supplementary_material1_aanabitarte.pdf]

Supplementary Material 1

2. Material and methods

2. 6. Description of the experiments

A basic experiment was carried out in each of four Gipuzkoan schools to test for the effect of the exposure to activities in green vs. grey spaces on the school students' performance in multiple-choice computer-based tests (128 questions). Students (random factor <Subject>) were randomly assigned to each of two levels of the fixed factor <Exposure>, that is <grey> and <green> spaces. Five univariate responses (Table 2) were recorded in two occasions on each subject, one before applying the exposure (level <before> of the fixed factor <Time>) and another after applying the exposure (level <after> of the fixed factor <Time>). Student's sex and age were also recorded.

2. 7. Repeated measures: statistical design and model

Each basic experiment is to be understood as a split-unit (or split-plot) statistical design (Casella 2008), where subjects represent the whole units, exposures represent the whole-unit treatments, and time represents the split-unit treatment. The two measures of each response taken on each subject are correlated although, there being just two repeated measures, then we may assume equicorrelation (Casella 2008) and, therefore, Cochran's theorem (Cochran 1934) is satisfied, with the final consequence that *F*-tests are valid. This specific type of split-unit design is known as a repeated-measures design and the corresponding statistical model may be written as follows (Pinheiro & Bates 2000; Casella 2008):

(equation 1) $$Y_{ijk} = \mu + \tau_i + \varepsilon_{ij} + \gamma_k + (\tau\gamma)_{ik} + \delta_{ijk},$$

where Y_{ijk} is a given response to Exposure i (= 1, …, t) of subject (whole unit) j (= 1, …, r) at Time k (= 1, …, g); μ is the overall mean effect; τ_i is the effect of Exposure i (i.e. the whole unit treatment: Subjects in Exposure); ε_{ij} is the effect of Subject j in Exposure i (i.e. the whole unit error), where ε_{ij} is

independently and identically distributed as $N(0, \sigma^2_\varepsilon)$; γ_k is the effect of Time k (i.e. the split unit treatment); $(\tau\gamma)_{ik}$ is the interaction between Exposure i and Time k; and δ_{ijk} is the experimental error (i.e. the split unit error: Time x Subjects in Exposure), where δ_{ijk}, which is independent of ε_{ij}, is assumed to be independently and identically distributed as $N(0, \sigma^2_\delta)$. Since Exposure has two levels (green/blue and grey spaces) and since Time has two levels (before and after), $t = 2$ and $g = 2$; since different numbers of subjects were used in each school, r varies in each basic experiment as specified in Table 1.

In a repeated-measures design, treatment (fixed factor <Exposure>, in this case) and time (treated as a fixed factor <Time>) are crossed (Casella 2008), which is a feature that repeated-measures designs share with two-way completely randomised designs. These two designs, however, differ in that randomization in completely randomised designs is not restricted, whereas in split-unit designs randomization is restricted to the whole units (i.e. the subjects). As a consequence, in a repeated-measures design there is a correlation structure that arises from the multiple observations taken on whole units (Pinheiro & Bates 2000), and this is the reason why we need equation 1 to describe the experimental structure. Repeated-measures designs also differ from simple (with just two levels of a given treatment) crossover designs, because in crossover designs each group of subjects receives both treatment levels, albeit in opposite orders (Casella 2008), whereas in repeated-measures designs each group of subjects receives just one treatment level. Thus, the effect of the exposure under a repeated-measures design is measured by the term $(\tau\gamma)_{ik}$ in equation 1, i.e. by the interaction between Exposure i and Time k. Since the goal of each of our basic experiments was to test for the effect of the exposure to activities in green/blue vs. grey spaces on the school students' performance in multiple-choice tests, we focused our analysis on the said term.

2. 8. Linear mixed effects modelling: the analysis of the basic experiments

Univariate responses from experimental settings may be analysed using the ANOVA approach (Casella 2008), but we preferred the Restricted Maximum Likelihood (ReML) approach within the

framework of linear mixed effects modelling (Pinheiro & Bates 2000; Bolker & Brooks 2009). This was so because the flexible ReML approach yields unbiased estimates for random terms in mixed models (such as the terms ε_{ij} and δ_{ijk} in equation 1), is resistant to unequal numbers of experimental subjects (as it occurs in most of our experiments: Table 1) and, if needed, allows modelling observed heterogeneity, thereby obtaining correct tests without transformation of the response variables (Pinheiro & Bates 2000; Madsen & Thyregod 2010). We used function lme() of R package nlme (Pinheiro, Bates et al. 2018) in R software v. 4.0.0 (R Core Team 2020).

2.9. Meta-analysis: combining the results of the basic experiments

Once we quantified the effect of the exposure levels in each basic experiment (as measured by the term $(\tau\gamma)_{ik}$ in equation 1), we combined the available evidence from each school using the meta-analysis methodology (Higgins, Thomas, et al., 2019). For this purpose, we used the function metagen() of the R package meta (Balduzzi, Rücker et al., 2019), applying the generic inverse variance method (Borenstein et al., 2010) for pooling the available data of each of the five response variables.

Because we were unready to assume that the effects from the basic experiments came from a homogeneous population (an assumption under the fixed effect model), we fitted both fixed (equation 2) and random (equation 3) effects models (Borenstein et al., 2010):

(equation 2) $$\hat{\theta}_k = \theta + \sigma_k \varepsilon_k,\ \epsilon_k \overset{i.i.d.}{\approx} \sim N(0,1);$$

(equation 3) $$\hat{\theta}_k = \theta + \mu_k + \sigma_k \varepsilon_k,\ \epsilon_k \overset{i.i.d.}{\approx} \sim N(0,1);\ \mu_k \overset{i.i.d.}{\approx} \sim N(0,\tau^2);$$

where ϑ_k and σ_k denote the intervention effect of experiment k and Var(ϑ_k), respectively, and the μ_k parameter allows for modelling a fraction of the observed between-experiment variability as an extra random effect.

In the latter case, between-experiment variance, τ^2, was estimated, as advised by Veroniki et al. (2016), via the restricted maximum-likelihood estimator (Viechtbauer, 2005). Notice that the fixed effects model is the particular case of the random effects model when $\tau^2 = 0$. Prediction intervals for each response variable were calculated according to Higgins et al. (2009) and presented according to Guddat et al. (2012). Finally, we used funnel plots (Sterne & Egger, 2001) and Egger et al.'s test (Egger, Smith, et al. 1997) to examine potential bias in the above meta-analyses.

5. References

Balduzzi, S., G. Rücker, et al. (2019). How to perform a meta-analysis with R: a practical tutorial. *Evidence Based Mental Health* 22(4): 153-160.

Barton, K. 2018. *MuMIn: Multi-Model Inference*, R package version 1.40.4.

Bolker, B. M., M. E. Brooks, et al. 2009. Generalized linear mixed models: a practical guide for ecology and evolution. *Trends in Ecology & Evolution* 24(3): 127-135.

Borenstein, M., L. V. Hedges, et al. (2010). A basic introduction to fixed-effect and random-effects models for meta-analysis. *Research Synthesis Methods* 1(2): 97-111.

Casella, G. 2008. *Statistical Design*. New York, Springer.

Cochran, W. G. 1934. The Distribution of Quadratic Forms in a Normal System with Applications to the Analysis of Covariance. *Proc. Cam. Phil. Soc*. 30: 178-191.

Davidian, M. and D. M. Giltinan. 1995. *Nonlinear Mixed Effects Models for Repeated Measurement Data*. Boca Raton, CRC Press. Chapman and Hall.

Egger, M., G. D. Smith, et al. 1997. Bias in meta-analysis detected by a simple, graphical test. *BMJ* 315(7109): 629-634.

Fox, J. & S. Weisberg. 2018. Visualizing Fit and Lack of Fit in Complex Regression Models with Predictor Effect Plots and Partial Residuals. *Journal of Statistical Software* 87(9): 1-27.

Guddat, C., U. Grouven, et al. 2012. A note on the graphical presentation of prediction intervals in random-effects meta-analyses. *Systematic reviews* 1: 34-34.

Higgins, J. P. T., S. G. Thompson, et al. (2009). A re-evaluation of random-effects meta-analysis. *Journal of the Royal Statistical Society: Series A* 172(1): 137-159.

Higgins, J. P. T., J. Thomas, et al., Eds. 2019. *Cochrane Handbook for Systematic Reviews of Interventions.* Chichester (UK), John Wiley & Sons.

Lüdecke, D. 2018. *sjPlot: Data Visualization for Statistics in Social Science*, R package version 2.6.2.

Madsen, H. and P. Thyregod. 2010. *Introduction to General and Generalized Linear Models*. Boca Raton, CRC Press. Chapman & Hall.

Pinheiro, J. J. and D. M. Bates. 2000. *Mixed-Effects Models in S and S-PLUS*, Springer.

Pinheiro, J. J., D. M. Bates, et al. 2018. *nlme: Linear and Nonlinear Mixed Effects Models*, R package version 3.1-137.

R Core Team. 2020. R: *A language and environment for statistical computing*, version 4.0.0. R Foundation for Statistical Computing. Vienna, Austria.

Sterne, J. A. C. & M. Egger. 2001. Funnel plots for detecting bias in meta-analysis: Guidelines on choice of axis. *Journal of Clinical Epidemiology* 54(10): 1046-1055.

Veroniki, A. A., D. Jackson, et al. (2016). Methods to estimate the between-study variance and its uncertainty in meta-analysis. *Research Synthesis Methods* 7(1): 55-79.

Viechtbauer, W. (2005). Bias and Efficiency of Meta-Analytic Variance Estimators in the Random-Effects Model. *Journal of Educational and Behavioral Statistics* 30(3): 261-293.
